# Substitution of linoleic acid with α-linolenic acid or long chain n-3 polyunsaturated fatty acid prevents Western diet induced nonalcoholic steatohepatitis

**DOI:** 10.1038/s41598-018-29222-y

**Published:** 2018-07-19

**Authors:** Sugeedha Jeyapal, Suryam Reddy Kona, Surekha Venkata Mullapudi, Uday Kumar Putcha, Puvaneswari Gurumurthy, Ahamed Ibrahim

**Affiliations:** 1 Department of Lipid Chemistry, National Institute of Nutrition, Hyderabad, India; 2 Department of Pathology, National Institute of Nutrition, Hyderabad, India

## Abstract

Imbalance in the n-6 polyunsaturated fatty acids (PUFA) and n-3 PUFA in the Western diet may increase the risk of nonalcoholic fatty liver disease (NAFLD). This study investigates the impact of substitution of linoleic acid with α-linolenic acid (ALA) or long chain (LC) n-3 PUFA and hence decreasing n-6:n-3 fatty acid ratio on high fat, high fructose (HFHF) diet induced nonalcoholic steatohepatitis (NASH). Male Sprague-Dawley rats were divided into four groups and fed control diet, HFHF diet (n-6:n-3 ratio of 200), HFHF diet with ALA (n-6:n-3 ratio of 2) or HFHF diet with LC n-3 PUFA (n-6:n-3 ratio of 5) for 24 weeks. Rats fed HFHF diet with n-6:n-3 ratio of 200 resulted in hepatic steatosis, induced glucose intolerance, insulin resistance and oxidative stress accompanied by increase in markers of inflammation, plasma lipids and aminotransferase levels. Histopathological examination of liver further confirmed the establishment of NASH. ALA and LC n-3 PUFA supplementation prevented hepatic steatosis and dyslipidemia by inhibiting lipogenesis and increasing insulin sensitivity. Furthermore, n-3 PUFA supplementation attenuated hepatic oxidative stress by restoring antioxidant status, decreased inflammation and preserved hepatic architecture. These finding suggest that decreasing n-6:n-3 ratio prevented HFHF induced NASH by attenuating oxidative stress and inflammation.

## Introduction

Nonalcoholic fatty liver disease (NAFLD) is the chronic liver disease of unknown etiology and is more common in affluent countries, affecting both adults and children. Clinically, it is defined as the fat accumulation in the hepatocytes (≥5% of the total weight) in the absence of excessive alcohol consumption (>20 g/d for women and >30 g/d for men)^1^. NAFLD is characterized by ectopic fat accumulation in hepatocytes (steatosis), which is relatively benign. However, approximately 20% of the individuals with simple steatosis develop nonalcoholic steatohepatitis (NASH) which may further progress into liver fibrosis, cirrhosis and eventually hepatocellular carcinoma^2,3^. By 2020, NASH has been projected to become the leading cause of liver transplantation, overtaking hepatitis C virus infection related transplantation in Western countries^4^. NAFLD is now considered as multisystem disease and associated with a range of chronic diseases such as cardiovascular disease, chronic kidney disease, type 2 diabetes and colorectal cancer^5^. It is estimated that, 20–30% of the general adult population is affected by NAFLD in Western countries^6^. The prevalence of NAFLD in India ranges from 16–32% in general population with higher prevalence in those with obesity, overweight and diabetes^7,8^. Recently, a “multiple parallel-hit” hypothesis has been proposed to explain the development and progression of NAFLD, which suggests that multiple insults such as insulin resistance, mitochondrial dysfunction, endotoxemia, adipocytokines, and gut microbiota synergistically act together resulting in immune cell activation leading to hepatic injury and cell death^9^.

Despite high prevalence of NAFLD, currently there is no FDA approved drug specifically for the treatment of NAFLD^10^. Hence, lifestyle modifications which include increased physical activity and proper diet are the only options for the management of NAFLD. Consumption of Western diet which are high calorie diets rich in processed sugars (fructose or sucrose) and fat has been implicated in the recent increase in the prevalence of NAFLD^11–13^. Furthermore, Kechagias *et al*. demonstrated that healthy subjects consuming fast food based diet for 4 weeks induced fatty liver, insulin resistance and increased aminotransferase levels^14^. Although Western diets are known to induce NAFLD, it is not clear how the individual components of the Western diet influence the development/progression of NAFLD. In this context, our recent study demonstrated that in a series of high energy diets mimicking Western diet, the type of dietary fat determines the development of advanced form of NAFLD^15^. Fructose in combination with saturated fatty acids (SFA) or *trans* fatty acids induced hepatic steatosis of comparable degree, whereas fructose in combination with *trans* fatty acids induced NASH with fibrosis^15^.

The polyunsaturated fatty acids (PUFA), linoleic acid (LA, 18:2 n-6) and α-linolenic acid (ALA, 18:3 n-3) are the short chain precursors that are converted into biologically active long chain (LC) PUFA such as arachidonic acid (20:4 n-6), eicosapentaenoic acid (EPA, 20:5 n-3) and docosahexaenoic acid (DHA, 22:6 n-3) respectively. The n-6 and n-3 PUFA are not interconvertible and are metabolically and functionally distinct^16^. Generally, the eicosanoids derived from n-6 PUFA are proinflammatory, whereas those derived from n-3 PUFA are anti-inflammatory. The PUFA composition of the cell membrane is determined by the dietary levels of n-6 and n-3 PUFA and their ratio. High intake of n-6 PUFA create proinflammatory milieu, which in turn, may affect the development or progression of several diet related chronic diseases including NAFLD^17^. In the present Western diet, the level of n-6 PUFA is significantly higher than the n-3 PUFA, which resulted in a shift of n-6:n-3 ratio to >10:1 from the traditional 2:1^18^. In fact, a study evaluating dietary pattern in patients with NASH, revealed high intake of n-6 PUFA and n-6:n-3 ratio thereby implicated in promoting NASH^19^. Clinical studies reported altered PUFA metabolism with reduced LC PUFA content in the liver of NASH patients suggesting unbalanced n-6 and n-3 PUFA in the pathogenesis of NASH^20–22^. Several animal studies showed that LC n-3 PUFA supplementation confer protection against the development of NAFLD in diet induced models of NAFLD^23–25^. Furthermore, transgenic mice engineered to express FAT-1 gene encoding n-3 fatty acid desaturase, which is capable of converting n-6 PUFA to LC n-3 PUFA prevents high fat^26^ and Western diet^27^ induced NAFLD by restoring LC n-3 PUFA. Nevertheless, clinical trials with LC n-3 PUFA supplementation in patients with NAFLD showed mixed results^28^. The failure of some of the clinical trials could be due to difference in the dosage of LC n-3 PUFA, duration of the trial, poor compliance or difference in the intake of other fatty acids. To our knowledge, there is no information about the level of n-3 PUFA (particularly in relation to n-6 PUFA) needed to prevent NAFLD. We hypothesize that imbalance in the dietary n-6:n-3 ratio in the Western diet is likely to induce NASH and substitution of n-6 PUFA with n-3 PUFA may prevent the development of NASH. Therefore, the objective of the present study was to investigate the impact of substituting n-6 PUFA (LA) with n-3 PUFA (ALA or LC n-3 PUFA) on the development of high fat, high fructose (HFHF) diet induced NASH. Blends of peanut oil, palmolein and linseed oil/fish oil were used to create a series of diets with differing levels of n-3 PUFA and ratios of n-6:n-3 fatty acids. The glucose metabolism, plasma lipid profile and adipocytokines, hepatic lipid content and histopathological changes were studied. In addition, the impact of n-3 PUFA supplementation on hepatic oxidative stress and expression of genes related to lipid metabolism and inflammation were also studied to understand the possible molecular mechanism by which n-3 PUFA supplementation prevents NASH.

## Materials and Methods

### Ethical approval

The present study was conducted as per the guidelines of the Committee for the Purpose of Control and Supervision of Experiments on Animals, Government of India. The research protocol was approved by institutional animal ethical committee of National Institute of Nutrition, Hyderabad, India (Approval ID-P4/IAEC/NIN/2012/4/AI/SD rats).

### Animal experiment and study design

Thirty-two weanling male Sprague-Dawley rats were acquired from animal house facility of National Institute of Nutrition, Hyderabad, India. Animals were housed individually in polypropylene cages with constantly regulated temperature (21 ± 1 °C) and light controlled housing conditions (12:12 h light/dark cycle). Rats were randomly divided into four groups (n = 8 per group) and fed starch/fructose - casein based synthetic diet. In the control group, starch was used as the source of carbohydrate, whereas, in the experimental groups, fructose was used as the source of carbohydrate. The n-6/n-3 PUFA ratio was altered by blending of peanut oil, palmolein and linseed oil (source of ALA) to get the ratio of 200 and 2 or by blending of peanut oil, palmolein and fish oil (source of EPA and DHA) to get the ratio of 5. The n-6:n-3 PUFA ratios of 2 and 5 were selected based on the results of our earlier studies on dietary PUFA and insulin resistance^29,30^. The various experimental groups were as follows: a control group with n-6/n-3 PUFA ratio of 200 (CON-200) and experimental groups with varying n-6/n-3 PUFA ratios of 200 (HFHF-200), 2 (HFHF-2) and 5 (HFHF-5). The diet composition was (% by weight) 50% carbohydrates, 20% casein, 20% oil, 5% cellulose, 1% vitamin mixture, 3.5% mineral mixture, 0.3% L-cysteine and 0.2% choline chloride. The salt and vitamin mixtures were prepared as per AIN-93 recommendations^31^. The HFHF diets also contain 1.5% cholesterol and 0.2% sodium cholate. All the diets were isocaloric, isolipidic and differed only in type of carbohydrate and n-6/n-3 PUFA ratio. Carbohydrates provided 44% of calories; fats provided 39% of calories and proteins provided 17% of calories. The total SFA, monounsaturated fatty acids (MUFA) and PUFA were similar in all the groups and n-6/n-3 ratio was altered by substituting n-6 PUFA with n-3 PUFA. Fatty acid analyses of the diets (Table 1) were performed by gas chromatography^32^. The diets were prepared once weekly by adding the oils to the base mixture containing other nutrients and stored at 4 °C before use. The oil and base mixture were stored at 4 °C until preparation of the diet. Fish oil (MAXEPA, Merck) was kept under an atmosphere of nitrogen and stored at −20 °C. All the diets were supplemented with α-tocopherol (0.015 g/kg diet) to prevent oxidation. Rats received food and drinking water ad libitum. Body weight was measured weekly and food consumption was monitored daily. At the end of 24 week, rats were fasted overnight and blood samples were collected from retro-orbital plexus in EDTA coated vials. Blood was centrifuged at 4,000 rpm for 15 min. at 4 °C and plasma was stored at −80 °C for subsequent determination of biochemical parameters. Rats were euthanized by CO_2_ asphyxiation. Liver, kidney, retroperitoneal fat (RP), epididymal fat (EP) and mesenteric fat (MS) were excised quickly and rinsed with saline. The organs were weighed, snap frozen in liquid nitrogen and stored at −80 °C for further analysis. A small piece of liver was stored in RNA Later (Sigma-Aldrich) for gene expression analysis and another portion of the liver was immediately immersed in neutral buffered 10% formalin for histopathological examination. Adiposity index was computed as the sum of visceral adipose tissue (RP + EP + MS) weights and expressed as a percentage of total body weight.

**Table 1:** Dietary fatty acid composition (g/100 g diet).

Fatty acid	CON-200	HFHF-200	HFHF-2	HFHF-5
∑ SFA	6.0	6.0	5.8	6.1
∑MUFA	7.9	7.9	7.6	8.0
∑PUFA	6.13	6.13	6.6	5.9
18:2n-6	6.1	6.1	4.4	4.9
18:3n-3	0.03	0.03	2.2	—
LCn-3PUFA	—	—	—	1.0
PUFA/SFA	1.0	1.0	1.1	1.0
n-6/n-3 ratio	200	200	2	5

### Intraperitoneal glucose tolerance test

The intraperitoneal glucose tolerance test (IPGTT) was performed as described previously^15^. Briefly, rats were fasted overnight and fasting blood sample was taken from retro-orbital plexus. The rats were injected intraperitoneally with a glucose solution (1 g/kg body weight). Blood samples were drawn at 15, 30, 60 and 120 min. intervals after the glucose injection and glucose levels were estimated by enzymatic kit method. Glucose and insulin areas under the curve (AUC) values were calculated using the trapezoidal rule.

### Plasma biochemistry

Glucose, triglycerides, total cholesterol, alanine aminotransferase (ALT) and aspartate aminotransferase (AST) levels in the plasma were estimated by kit method (Biosystems, Barcelona, Spain). Plasma insulin was measured using rat insulin ELISA kit (Mercodia AB, Uppsala, Sweden) according to the manufacturer’s instructions. Homeostasis model assessment of insulin resistance (HOMA-IR) index was calculated from fasting plasma glucose and insulin to estimate insulin resistance. The adiponectin (Invitrogen, California, USA), leptin (EMD Millipore, Billerica, MA, USA) and resistin (BioVendor, Czech Republic) levels in the plasma were estimated by commercial ELISA kits.

### Determination of liver triglyceride and cholesterol contents

Liver tissue (50–100 mg) was homogenized in 5% aqueous Nonidet P-40 (NP-40) solution to solubilize the lipids^15,33^. Intrahepatic triglycerides and cholesterol were assayed using enzymatic kit (Biosystems, Barcelona, Spain).

### Assessment of lipid peroxidation, activity of antioxidant enzymes and glutathione levels in the liver

Liver lipid peroxidation in the homogenate was estimated by measuring malondialdehyde using thiobarbituric acid reactive substances (TBARS) assay^34^. Catalase activity was measured according to the method of Aebi^35^. Glutathione peroxidase (GPX) activity was determined according to Flohe and Gunzler^36^. The total superoxide dismutase (SOD) activity was measured following the method of McCord and Fridovich^37^. Reduced glutathione content was measured by the Anderson method^38^. Total protein content of the liver homogenate was determined by Lowry’s method^39^.

### Quantification of liver phospholipid fatty acid composition

Liver total lipids were extracted according to Bligh and Dyer method^40^. To analyze liver phospholipid fatty acid composition, neutral lipids were separated from phospholipids as described previously^15^. Phospholipid fatty acid profile was quantified by gas chromatography as described^32^.

### Liver gene expression studies by quantitative real time PCR

RNA extraction and cDNA synthesis were performed as described previously^15^. Quantitative PCR was performed by using SYBR Green Master mix kit (Takara, Otsu, Shiga, Japan) in Bio-rad CFX96 system (Bio-rad Laboratories, Hercules, California, USA) with β-actin as the control gene. Primers were obtained from Sigma - Aldrich and the specific primer sequences used in this study are listed in Supplementary Table S1. Relative expression of each gene was calculated by the 2^–(ΔΔCt)^ method^41^.

### Histological assessment of liver

Liver samples fixed in formalin were embedded in paraffin wax and cut into 5 μm sections for hematoxylin and eosin (H&E) staining. Hepatic steatosis and fibrosis were assessed by Oil Red O staining and Masson’s trichrome staining respectively. NAFLD activity score (NAS) was calculated based on the individual scores of steatosis (0–3 points), lobular inflammation (0–3 points) and hepatocyte ballooning (0–2 points) in a blinded manner to assess the severity of NAFLD^42^. A score of >4 was considered as NASH.

### Statistical analysis

All the data were presented as mean ± SD. The differences between the groups were analyzed by one-way analysis of variance (ANOVA) with least significant difference (LSD) post hoc test using SPSS 16.0 software package. Significance was considered to be at P < 0.05.

## Results

### General characteristics

Food intake and body weight gain were comparable between the groups (Table 2). Compared to control diet (CON-200), the HFHF diet (HFHF-200) increased liver and kidney weights 1.4 (P < 0.001) and 1.2 (P < 0.001) folds respectively. However, substitution of n-6 PUFA with n-3 PUFA in HFHF fed rats (HFHF-2 and HFHF-5) significantly decreased liver weight without altering kidney weight. The weights of retroperitoneal and mesenteric fat pads were increased by 70% (P < 0.001) and 30% (P = 0.045) respectively in rats fed diet with HFHF compared to control diet fed rats. Accordingly, the calculated values of adiposity index were increased by 38% (P = 0.003) in rats fed diet with HFHF. Substitution of n-6 PUFA with n-3 PUFA normalized the fat pad weights and adiposity index.

**Table 2:** Effect of substitution of LA with ALA or LC n-3 PUFA on food intake, body weight gain and organ weights.

	CON-200	HFHF-200	HFHF-2	HFHF-5
Food intake (g/day)	15.1 ± 1.0^a^	14.5 ± 1.4^a^	15.4 ± 1.5^a^	14.7 ± 1.2^a^
Body weight gain (g)	335 ± 22.0^a^	308 ± 45.0^a^	331 ± 35.0^a^	317 ± 43.5^a^
Liver (% body weight)	2.4 ± 0.2^a^	3.4 ± 0.4^b^	3.0 ± 0.3^c^	2.9 ± 0.3^c^
Kidney (% body weight)	0.66 ± 0.03^a^	0.77 ± 0.03^bc^	0.75 ± 0.05^b^	0.79 ± 0.02^c^
Epididymal fat (% body weight)	1.1 ± 0.2^ab^	1.2 ± 0.3^a^	1.0 ± 0.1^ab^	0.9 ± 0.15^b^
Retroperitoneal fat (% body weight)	1.0 ± 0.22^ac^	1.7 ± 0.22^b^	1.2 ± 0.30^a^	0.7 ± 0.34^c^
Mesenteric fat (% body weight)	0.37 ± 0.06^ac^	0.48 ± 0.06^b^	0.47 ± 0.07^bc^	0.34 ± 0.14^a^
Adiposity Index (%)	2.4 ± 0.42^ac^	3.3 ± 0.46^b^	2.7 ± 0.41^c^	2.0 ± 0.60^a^

Results are expressed as mean ± SD, n = 6–8 per group.

Mean values within a row with different superscript letters were significantly different at p < 0.05. Statistical analyses between the groups were performed by one way ANOVA with LSD post hoc test.

### Substituting LA with ALA or LC n-3 PUFA improved HFHF induced alteration in fasting metabolic parameters

Compared to control group, the HFHF feeding increased plasma triglycerides and cholesterol levels by 39% (P = 0.011) and 47% (P < 0.001) respectively (Table 3). Substitution of n-6 PUFA with n-3 PUFA normalized the plasma lipid levels. Fasting plasma glucose was increased (13%, P = 0.011) in rats fed diet with HFHF and substitution of n-6 PUFA with n-3 PUFA normalized the level. Rats fed the HFHF diet had higher fasting plasma insulin level (by 1.9 fold, P < 0.001) and HOMA-IR (by 2.1 fold, P < 0.001) than those fed control diet. In HFHF fed rats, substituting n-6 PUFA with n-3 PUFA decreased (HFHF-2) or normalized (HFHF-5) the plasma insulin level and HOMA-IR. Rats fed the HFHF diet had lower plasma adiponectin (P < 0.001) and higher leptin (P = 0.027) compared to control diet fed rats. Substitution of n-6 PUFA with n-3 PUFA in HFHF diet normalized the leptin level without altering the adiponectin level. Furthermore, adiponectin:leptin ratio (P = 0.004) was lower in rats fed the HFHF diet compared to control diet fed rats and substitution of n-6 PUFA with n-3 PUFA normalized the ratio. Although plasma resistin level was not altered by HFHF diet, substitution of LA with ALA or LC n-3 PUFA in HFHF diet decreased the resistin levels by 15% (P = 0.033) and 30% (P < 0.001) respectively.

**Table 3:** Effect of substitution of LA with ALA or LC n-3 PUFA on plasma biochemical parameters.

	CON-200	HFHF-200	HFHF-2	HFHF-5
Triglycerides (mg/dL)	64 ± 11.0^a^	89 ± 21.3^b^	55 ± 8.1^a^	59 ± 17.3^a^
Total cholesterol (mg/dL)	75 ± 4.0^a^	110 ± 17.7^b^	68 ± 10.1^a^	66 ± 8.6^a^
Glucose (mg/dL)	102 ± 7.6^a^	115 ± 8.2^b^	98 ± 7.8^a^	96 ± 6.5^a^
Fasting insulin (mU/L)	7.0 ± 0.9^a^	13.4 ± 4.9^b^	10.3 ± 1.6^bc^	7.2 ± 0.7^ac^
HOMA-IR	1.7 ± 0.3^a^	3.6 ± 1.0^b^	2.5 ± 0.4^c^	1.9 ± 0.3^ac^
Adiponectin (μg/mL)	22 ± 2.1^a^	16 ± 3.9^b^	18 ± 2.7^b^	18 ± 2.5^b^
Leptin (pg/mL)	538 ± 49^a^	831 ± 266^b^	569 ± 307^a^	519 ± 116^a^
Adiponectin/leptin ratio	0.041 ± 0.007^a^	0.019 ± 0.007^bc^	0.031 ± 0.02^ac^	0.036 ± 0.007^a^
Resistin (ng/mL)	25.2 ± 2.01^ac^	26.0 ± 3.6^a^	22.0 ± 3.4^bc^	18.3 ± 4.1^b^

Results are expressed as mean ± SD, n = 6–8 per group.

Mean values within a row with different superscript letters were significantly different at p < 0.05. Statistical analyses between the groups were performed by one way ANOVA with LSD post hoc test.

### Substituting LA with ALA or LC n-3 PUFA prevented the HFHF induced glucose intolerance and insulin resistance

**Figure 1:**
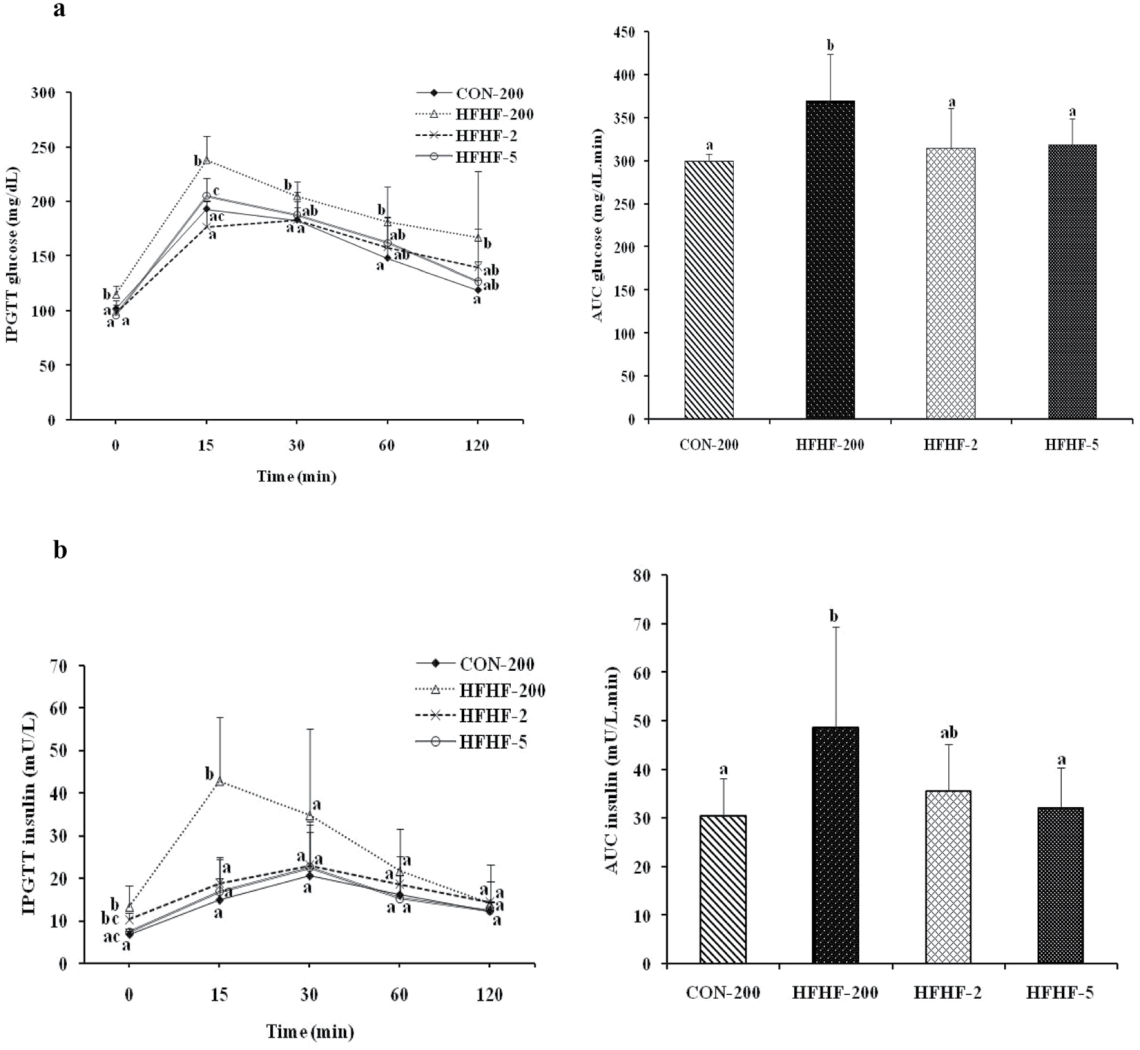
Effect of substitution of LA with ALA or LC n-3 PUFA on IPGTT and AUC of (**a**) plasma glucose and (**b**) plasma insulin. Results are expressed as mean ± SD, n = 6–8 per group. Mean values at a time or AUC mean with unlike letters significantly different at p < 0.05. Statistical analyses between the groups were performed by one way ANOVA with LSD post hoc test.

As depicted in the Fig. 1, HFHF feeding induced glucose intolerance as evidenced by high glucose excursion and AUC of glucose (23%, P = 0.006) in response to a glucose load in the IPGTT. In contrast, substitution of n-6 PUFA with n-3 PUFA prevented the HFHF induced glucose intolerance as evidenced by decreased glucose excursion and AUC of glucose (Fig. 1a). Along with impaired glucose intolerance, HFHF feeding induced insulinemia as evidenced by increased insulin AUC (60%, P = 0.023) after a glucose load and substitution of n-6 PUFA with n-3 PUFA corrected the HFHF induced insulinemia (Fig. 1b).

### Substituting LA with ALA or LC n-3 PUFA mitigated HFHF induced hepatomegaly and liver injury

**Figure 2:**
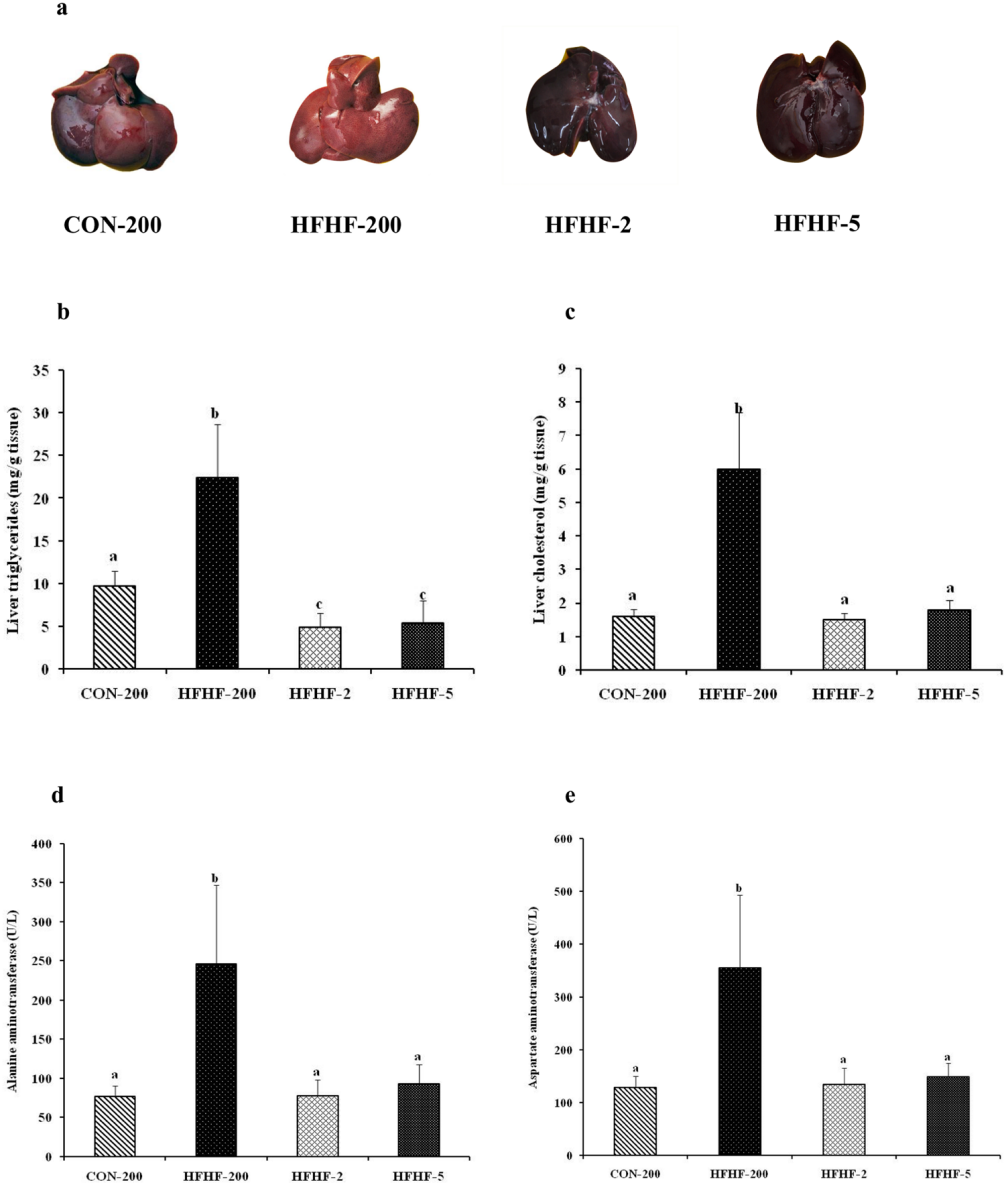
Effect of substitution of LA with ALA or LC n-3 PUFA on (**a**) liver morphology, (**b**) hepatic triglyceride, (**c**) hepatic cholesterol, (**d**) plasma ALT and (**e**) plasma AST. Results are expressed as mean ± SD, n = 6–8 per group. Mean values with unlike letters significantly different at p < 0.05. Statistical analyses between the groups were performed by one way ANOVA with LSD post hoc test.

As shown in Fig. 2, compared with the control group, the livers of the rats fed diet with HFHF were grossly enlarged and pale in color (Fig. 2a) combined with accumulation of triglycerides and cholesterol (Fig. 2b,c). Nevertheless, substitution of n-6 PUFA with n-3 PUFA reduced hepatomegaly and lipid accumulation. The activities of plasma ALT and AST, which are the markers of hepatic injury, were increased by 3.2 fold (P < 0.001) and 2.8 fold (P < 0.001) respectively in the rats fed diet with HFHF, compared to control diet fed rats (Fig. 2d,e). Substitution of n-6 PUFA with n-3 PUFA normalized the aminotransferase activity.

### Substituting LA with ALA or LC n-3 PUFA attenuate hepatic oxidative stress and increase antioxidant enzyme activity and glutathione level in HFHF fed rat

**Figure 3:**
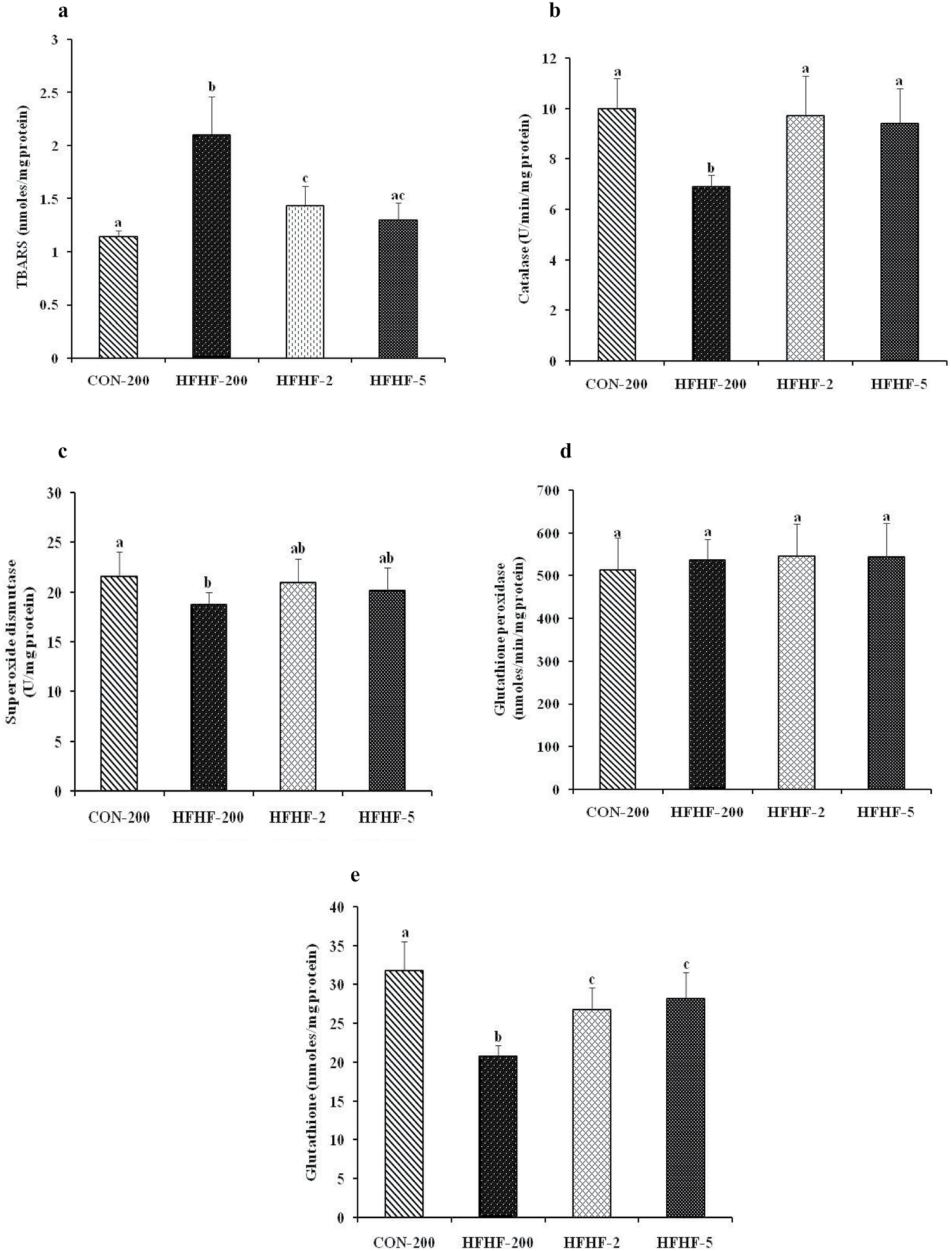
Effect of substitution of LA with ALA or LC n-3 PUFA on liver oxidative stress and antioxidant enzyme activity. (**a**) TBARS level (**b**) catalase activity (**c**) superoxide dismutase activity (**d**) glutathione peroxidase activity and (**e**) glutathione level. Results are expressed as mean ± SD, n = 6–8 per group. Mean values with unlike letters significantly different at p < 0.05. Statistical analyses between the groups were performed by one way ANOVA with LSD post hoc test.

Liver TBARS, which is the marker of oxidative stress was increased by 1.8 fold (P < 0.001) in HFHF fed rats compared with the rats fed control diet (Fig. 3a). In HFHF fed rats, substituting n-6 PUFA with n-3 PUFA decreased (HFHF-2) or normalized (HFHF-5) the TBARS level. In addition, catalase, superoxide dismutase activities and glutathione level were decreased by 1.4 fold (P < 0.001), 1.2 fold (P = 0.032) and 1.5 fold (P < 0.001) respectively in the rats fed HFHF diet when compared with rats fed control diet (Fig. 3b–e). However, substitution of n-6 PUFA with n-3 PUFA restored the antioxidant enzyme activities and glutathione levels in the liver.

### Substituting LA with ALA or LC n-3 PUFA restored the hepatic expression of genes involved in lipid metabolism and proinflammatory cytokines in HFHF fed rat

**Figure 4:**
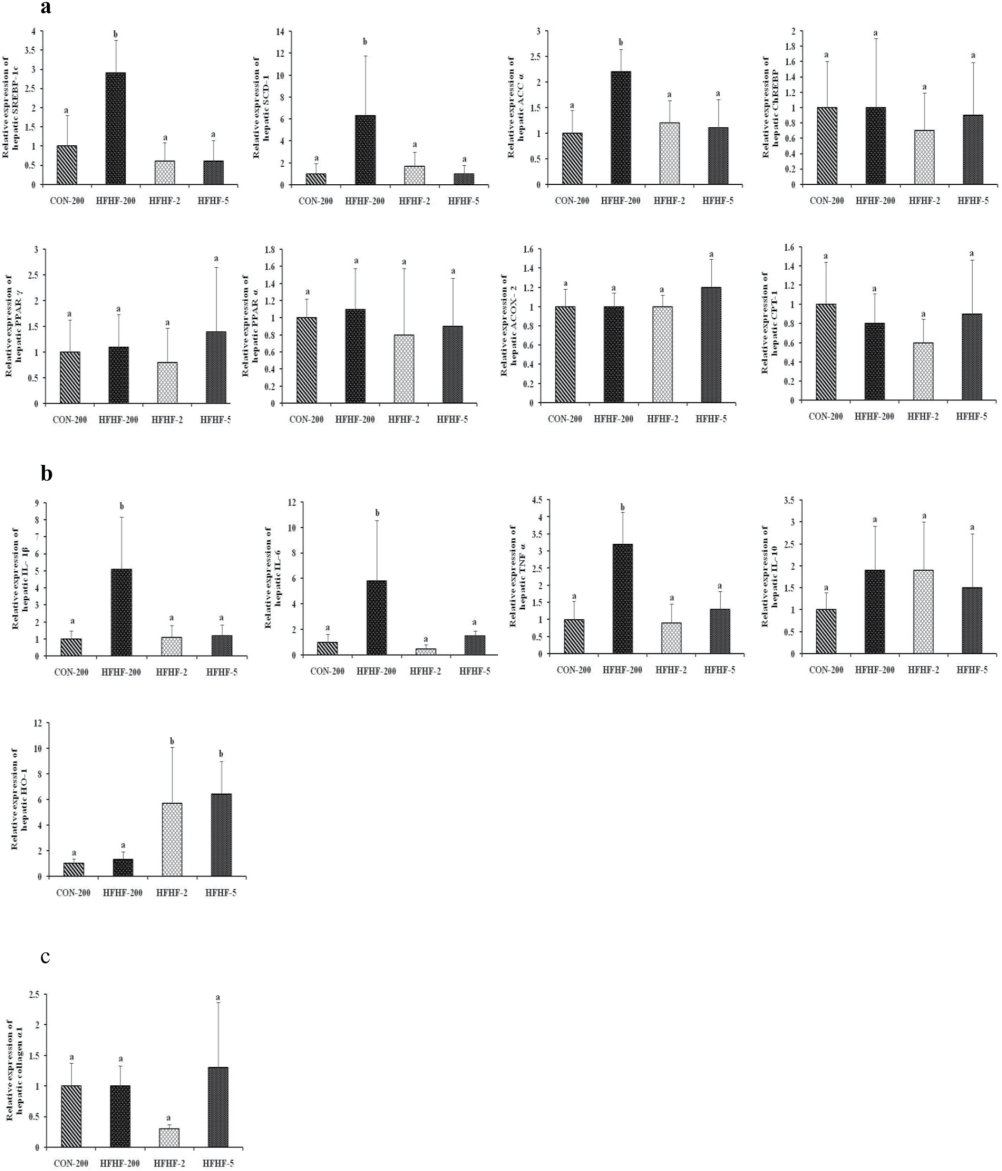
Effect of substitution of LA with ALA or LC n-3 PUFA on relative mRNA expression of genes involved in (**a**) lipid metabolism such as Sterol regulatory element binding protein-1c (SREBP-1c), Stearoyl-CoA desaturase-1 (SCD-1), Acyl CoA carboxylase-α (ACC-α), Carbohydrate-responsive element binding protein (ChREBP), Peroxisome proliferator activated receptor-γ (PPAR-γ), Peroxisome proliferator activated receptor-α (PPAR-α), Acyl CoA oxidase-2 (ACOX-2) and Carnitine palmitoyltransferase-1 (CPT-1) (**b**) Inflammation such as Interleukin-1β (IL-1β), Interleukin-6 (IL-6), Tumor necrosis factor-α (TNF-α), Interleukin-10 (IL-10) and Heme oxygenase (HO-1) and (**c**) fibrosis such as Collagen α1. Results are expressed as mean ± SD, n = 4 per group. Mean values with unlike letters significantly different at p < 0.05. Statistical analyses between the groups were performed by one way ANOVA with LSD post hoc test.

To understand the molecular mechanisms by which n-3 PUFA supplementation protects the HFHF induced development of NASH, the mRNA expression levels of genes associated with lipid metabolism, inflammation and fibrosis were analyzed. As shown in Fig. 4, HFHF feeding upregulated the expression of SREBP-1c (2.9 fold, P = 0.002), the key transcriptional regulator of *de novo* lipogenesis and its target genes SCD-1 (6.3 fold, P = 0.023) and ACC α (2.2 fold, P = 0.005), whereas, the expression of other lipogenic genes, ChREBP and PPAR γ were not altered (Fig. 4a). The mRNA levels of PPAR α, ACOX-2 and CPT-1, which are involved in fatty acid oxidation, were not altered by feeding HFHF diet. Substitution of n-6 PUFA with n-3 PUFA in HFHF diet normalized the mRNA levels of SREBP-1c, SCD-1 and ACC α. The HFHF feeding induced inflammation as shown by increased hepatic mRNA expression level of proinflammatory cytokines, TNFα, IL-1β and IL-6 by 3.2 fold (P = 0.001), 5.1 fold (P = 0.004) and 5.8 fold (P = 0.016) respectively (Fig. 4b). Substitution of n-6 PUFA with n-3 PUFA in HFHF diet normalized the proinflammatory cytokine expression levels. Interestingly, substitution of n-6 PUFA with n-3 PUFA in HFHF diet upregulated the mRNA expression level of HO-1 (Fig. 4b). Neither HFHF feeding nor n-3 PUFA supplementation altered the mRNA expression level of anti-inflammatory cytokine (IL-10) and fibrogenic marker (collagen α1) (Fig. 4c).

### Substituting LA with ALA or LC n-3 PUFA attenuated liver pathology in HFHF induced NASH

**Figure 5:**
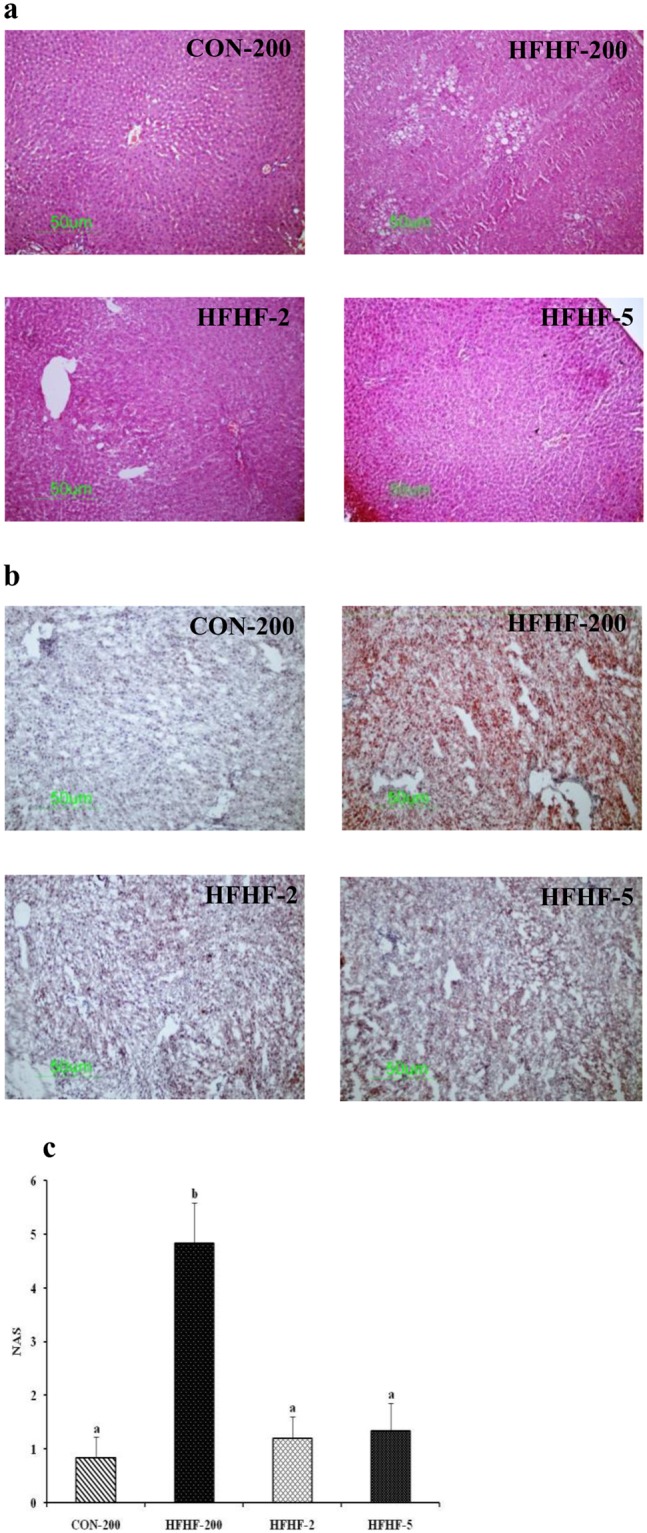
Effect of substitution of LA with ALA or LC n-3 PUFA on liver histology and NAS. Representative liver histology with (**a**) H & E staining (100X) and (**b**) Oil red O staining (100X). (**c**) NAS. Results are expressed as mean ± SD, n = 6 per group. Mean values with unlike letters significantly different at p < 0.05. Statistical analyses between the groups were performed by one way ANOVA with LSD post hoc test.

Representative histological finding and NAS in the livers of the rat fed various diets are shown in Fig. 5. The liver H&E staining of control rat showed normal parenchyma and architecture without any evidence of steatosis or inflammation. In contrast, the HFHF feeding induced histological features of NASH with microvesicular and macrovesicular steatosis, infiltration of inflammatory cells and hepatocyte ballooning (Fig. 5a), leading to 5.8 fold (P < 0.001) increased NAS (Fig. 5c). Oil red O staining showed extensive lipid deposition in hepatocytes from rats fed HFHF diet (Fig. 5b). Masson trichrome staining showed no evidence of fibrosis. Substitution of n-6 PUFA with n-3 PUFA in HFHF diet reduced fat deposition, inflammation and ballooning, resulting in normalization of NAS (Fig. 5a–c).

### Liver phospholipid fatty acid profile

In line with the liver SCD-1 mRNA expression level, the data on phospholipid fatty acid composition showed that compared to control, HFHF feeding increased the levels of C16:1 and C18:1 with consequent increase in the C16:1/C16:0 (2.3 fold, P < 0.001) and C18:1/C18:0 (1.4 fold, P = 0.003) ratios which are the estimated index of SCD-1 activity (Table 4). However, substitution of n-6 PUFA with n-3 PUFA normalized the ratios. Total SFA, MUFA and PUFA did not differ among the groups. Decreasing n-6:n-3 ratio in HFHF group significantly altered the n-6 and n-3 PUFA composition in phospholipids. Substituting n-6 PUFA with n-3 PUFA in the diet was associated with decrease in the proportion of LC n-6 PUFA (C20:4 n-6, C22:4 n-6 and C22:5 n-6) and increase in the proportion of LC n-3 PUFA (C20:5 n-3, C22:5 n-3 and C22:6 n-3) in the liver phospholipids. Compared to ALA supplementation (HFHF-2), LC n-3 PUFA supplementation (HFHF-5) increased the incorporation of C20:5 n-3 and C22:6 n-3 to a greater extent.

**Table 4:** Effect of substitution of LA with ALA or LC n-3 PUFA on liver phospholipid fatty acid composition.

FA (nmole%)	CON-200	HFHF-200	HFHF-2	HFHF-5
C14:0	0.24 ± 0.03^a^	0.29 ± 0.06^a^	0.18 ± 0.05^b^	0.18 ± 0.02^b^
C16:0	21 ± 0.84^a^	22 ± 1.54^a^	22 ± 1.24^a^	24 ± 0.9^b^
C16:1	0.16 ± 0.02^a^	0.40 ± 0.16^b^	0.27 ± 0.05^a^	0.26 ± 0.04^a^
C16:1/C16:0	0.008 ± 0.001^a^	0.018 ± 0.006^b^	0.012 ± 0.002^a^	0.011 ± 0.002^a^
C18:0	27 ± 0.94^a^	25 ± 0.96^b^	25 ± 1.2^b^	24 ± 0.95^b^
C18:1 *cis*	6.6 ± 1.2^a^	8.5 ± 1.5^b^	6.8 ± 0.77^a^	6.5 ± 0.54^a^
C18:1/C18:0	0.24 ± 0.05^a^	0.34 ± 0.06^b^	0.28 ± 0.04^a^	0.27 ± 0.03^a^
C18:2 *n-6*	9.6 ± 0.97^a^	12.0 ± 1.4^b^	14.1 ± 1.2^c^	12.6 ± 0.23^b^
C18:3 *n-3*	—	—	0.24 ± 0.03	—
C20:4 *n-6*	30 ± 0.88^a^	27 ± 1.42^b^	18 ± 0.75^c^	15 ± 1.86^d^
C20:5 *n-3*	—	—	2.3 ± 0.35^a^	2.8 ± 0.43^b^
C22:4 *n-6*	0.73 ± 0.1^a^	0.51 ± 0.08^b^	0.30 ± 0.04^c^	0.32 ± 0.09^c^
C22:5 *n-6*	1.83 ± 0.19^a^	0.60 ± 0.20^b^	—	—
C22:5 *n-3*	0.21 ± 0.05^a^	0.19 ± 0.03^a^	1.65 ± 0.2^b^	1.38 ± 0.06^c^
C22:6 *n-3*	2.30 ± 0.12^a^	3.20 ± 0.4^b^	8.93 ± 0.66^c^	12.1 ± 0.28^d^
ΣSFA	48.5 ± 1.15^a^	47.3 ± 1.30^a^	47.2 ± 1.14^a^	48.2 ± 1.0^a^
ΣMUFA	7.1 ± 1.05^a^	7.9 ± 1.05^a^	7.0 ± 0.82^a^	7.1 ± 0.81^a^
ΣPUFA	44.8 ± 0.71^a^	44.6 ± 2.0^a^	45.8 ± 0.93^a^	44.7 ± 0.7^a^
LC*n-6* PUFA	33.0 ± 0.92^a^	28.3 ± 1.5^b^	18.5 ± 0.8^c^	15.2 ± 1.8^d^
LC*n-3* PUFA	2.5 ± 0.12^a^	3.4 ± 0.47^b^	13.0 ± 0.6^c^	16.2 ± 0.48^d^
LC*n-6*/*n-3*	13.2 ± 0.4^a^	8.8 ± 0.9^b^	1.44 ± 0.11^c^	0.97 ± 0.11^c^

Σ LC n-6 PUFA = C20:4 n-6 + C22:4 n-6 + C22:5 n-6, Ʃ LC n-3 PUFA = C20:5 n-3 + C22:5 n-3 + C22:6 n-3.

Results are expressed as mean ± SD, n = 6–8 per group. Mean values within a row with different superscript letters were significantly different at p < 0.05. Statistical analyses between the groups were performed by one way ANOVA with LSD post hoc test.

## Discussion

High levels of n-6 PUFA in the Western diet could potentiate inflammatory response and may induce NASH. In the present study by mimicking typical Western diet, we investigated the impact of substitution of n-6 PUFA with n-3 PUFA (varying n-6:n-3 ratio) on the development of NASH. The n-6:n-3 ratios were chosen on the basis of our previous studies, wherein we have investigated the impact of varying n-6:n-3 ratios of 200, 50, 10, 5 and 2, representing wide range of n-6 and n-3 PUFA in the present human diet, on sucrose induced insulin resistance^29,30^. The results of the aforementioned studies demonstrated that compared to n-6:n-3 ratios of 200, 50 and 10, substitution of LA with ALA (n-6:n-3 ratio of 2) or LC n-3 PUFA (n-6:n-3 ratio of 5) prevented sucrose induced insulin resistance and dyslipidemia. Hence, n-6:n-3 ratios of 200 (representing high n-6 PUFA and n-3 PUFA deficient), 2 and 5 were selected. The results of the study, for the first time, demonstrated that partial replacement of LA with ALA (n-6: n-3 ratio of 2) or LC n-3 PUFA (n-6:n-3 ratio of 5) attenuated Western diet induced NASH. The protective effect of n-3 PUFA supplementation include attenuation of insulin resistance and glucose intolerance, optimization of plasma and liver lipid levels, mitigation of hepatic oxidative stress and inflammation, and improvements of aminotransferase activities and histological score.

It is established that NAFLD is the hepatic manifestation of metabolic syndrome and insulin resistance may play a key role in the development and progression of NAFLD^43^. However, whether insulin resistance is the cause or consequence of NAFLD is yet to be established^44^. Elevated levels of aminotransferase, which is the marker of liver injury has been reported in NASH subjects with insulin resistance, suggesting that insulin resistance may be involved in the progression of NAFLD^45,46^. Increased visceral adiposity by facilitating adipose tissue lipolysis and ectopic fat accumulation induces insulin resistance. Although obesity is the major risk factor for NAFLD, it is not rare in individuals with normal BMI particularly in Asian population^47^. Several animal studies demonstrated that both LC n-3 PUFA and ALA prevented high fat or high sucrose diet induced insulin resistance by reducing visceral adiposity^48,49^. Our earlier studies also showed that substitution of linoleic acid with ALA (n-6:n-3 ratio of 2) or LC n-3 PUFA (n-6:n-3 ratio of 5) prevented sucrose induced insulin resistance by increasing peripheral insulin sensitivity^29,30^. In the present study, rats fed HFHF diet increased visceral adiposity without altering the body weight. Also, substitution of n-6 PUFA with n-3 PUFA in HFHF diet effectively prevented the insulin resistance and glucose intolerance. The protective effect of n-3 PUFA supplementation was associated with decrease in visceral adiposity.

High level of fructose and n-3 PUFA deficiency in the Western diet has been implicated in the development of NAFLD^50^. Hepatic lipogenesis is modulated by SREBP-1c, which is the key transcription factor of *de novo* lipogenesis and regulates downstream genes such as SCD-1, ACC and FAS. SREBP-1c plays a crucial role in the development of NAFLD^51,52^. Indeed, Yamada *et al*. demonstrated that in patients with NASH, the gene expression of hepatic SREBP-1, SCD-1, FAS and PPARγ were enhanced^53^. In addition, C18:1/C18:0 ratio was associated with the steatosis score whereas C16:1/C16:0 ratio was associated with lobular inflammation score^53^. Several studies have shown that the fructose component of the Western diet is responsible for the *de novo* lipogenesis by up regulating SREBP-1c^54^. It is well documented that dietary n-3 PUFA prevents hepatic steatosis by down regulating SREBP-1c and up regulating PPAR-α which regulates genes involved in fatty acid oxidation^55,56^. In the present study, HFHF feeding induced hepatic steatosis by up regulating the hepatic mRNA expression of SREBP-1c and SCD-1 without altering PPAR-α expression. Additionally, the desaturation index C16:1/C16:0 and C18:1/C18:0, which is used to estimate the SCD-1 activity was significantly increased which further supports the involvement of SCD-1 in hepatic steatosis. Substitution of n-6 PUFA with n-3 PUFA (ALA or LC n-3 PUFA) in HFHF diet prevented hepatic steatosis by down regulating the expression of SREBP-1c and SCD-1. Decreased C18:1/C18:0 and C16:1/C16:0 ratios in liver by n-3 PUFA supplementation further confirms the anti-steatotic and anti-inflammatory role of n-3 PUFA. Studies have shown that among the LC n-3 PUFA, DHA is the potent regulator of SREBP-1c, whereas EPA is PPAR- α activator^55^. The observed anti-steatotic effect of ALA supplementation could be due to its conversion to DHA and EPA. Recently, Monteiro *et al*. demonstrated that using delta-5 and delta-6 knockout mice, ALA supplementation prevents hepatic lipogenesis and the development of steatosis^57^, suggesting that ALA could bring about its anti-steatotic effect which is independent of its conversion to LC n-3 PUFA.

Besides significant reduction in hepatic steatosis, n-3 PUFA supplementation also prevents HFHF induced dyslipidemia. Several animal and human studies suggest that dietary n-3 PUFA (ALA and LC n-3 PUFA) decrease the risk factors associated with metabolic syndrome including dyslipidemia^58,59^. It is worthy to mention that both ALA and LC n-3 PUFA are equally effective in preventing HFHF induced dyslipidemia.

According to multiple hit hypothesis of NAFLD pathogenesis, oxidative stress is considered as a key contributor to the transition from simple steatosis to NASH and fibrosis^60^. The excessive production of reactive oxygen species due to mitochondrial dysfunction causes lipid peroxidation which in turn induces inflammation and activation of stellate cells leading to fibrogenesis. Indeed, elevated levels of markers of oxidative stress and lipid peroxidation have been reported in patients with NASH^60^. In the present study, HFHF feeding induced oxidative stress as evidenced by increase in liver TBARS and decrease in antioxidant enzyme activity (catalase and SOD) and GSH. Due to high degree of unsaturation, there is a concern that n-3 PUFA particularly LC n-3 PUFA may induce oxidative stress and lipid peroxidation. However, emerging evidence indicate that although high levels of LC n-3 PUFA induce oxidative stress, moderate/appropriate dose of n-3 PUFA exerts antioxidant effect^61^. In fact, a recent study showed that LC n-3 PUFA supplementation decrease hepatic oxidative stress and triglyceride content in high fat diet induced fatty liver^25^. In the present study, both ALA and LC n-3 PUFA supplementation prevented the HFHF induced hepatic oxidative stress by improving the antioxidant status through restoring the antioxidant enzyme activities and GSH level. HO-1 is an inducible antioxidant enzyme and plays an important role in the cytoprotection and tissue injury^62^. Interestingly, n-3 PUFA supplementation up regulated the mRNA expression of HO-1 although HFHF feeding per se did not alter its expression. Recent *in vitro* studies have shown that LC n-3 PUFA prevents oxidative stress by up regulating HO-1 through activation of nuclear factor erythroid 2 related factor (Nrf-2)^63^. Further studies are necessary to understand the molecular mechanism by which LC n-3 PUFA modulates the antioxidant enzymes particularly in NAFLD. Oxidative stress triggers inflammatory process by activating redox - sensitive transcriptional factor, NF-κB thereby causing necroinflammation leading to NASH. Rats fed HFHF diet showed up regulation of proinflammatory cytokines with the development of NASH and n-3 PUFA supplementation has been shown to prevent NASH by suppressing the inflammatory response as evidenced by down regulation of proinflammatory cytokines. Tapia *et al*. showed the LC n-3 PUFA supplementation prevents high fat induced hepatic steatosis and inflammation by down regulating NF-κB^23^. Furthermore, a recent randomized clinical trial demonstrated that combined ALA and LC n-3 PUFA supplementation in NASH patients decreased plasma lipids with improvements in liver histology^64^. In addition to down regulating the proinflammatory cytokines, the anti-inflammatory effect of n-3 PUFA supplementation may also be mediated by resolvins and protectins derived from EPA and DHA which are known to resolve the inflammation^65^.

Dysregulation of adipocytokines by orchestrating proinflammatory and insulin resistance state may contribute to the development and progression of NAFLD. Leptin is known to induce insulin resistance, hepatic steatosis and has proinflammatory role and also contributes to fibrosis, whereas, adiponectin has anti-inflammatory and insulin sensitizing effect. Several clinical studies demonstrated that circulating leptin, TNF-α and IL-6 were significantly higher in patients with NAFLD/NASH, conversely adiponectin levels were significantly reduced in NAFLD/NASH patients^66^. However, the role of resistin which is proinflammatory in NAFLD is inconclusive. Lemoite *et al*. showed that in patients with NAFLD the adiponectin:leptin ratio is inversely related to the severity of NAFLD and proposed as predictive factor of NASH^67^. There is evidence that LC n-3 PUFA modulates the adipocytokines by increasing circulatory adiponectin and decreasing leptin levels^68^. Our study showed that both ALA and LC n-3 PUFA supplementation decreased plasma leptin and resistin levels and increased adiponectin:leptin ratio, suggesting that by correcting adipocytokine imbalance, n-3 PUFA ameliorates Western diet induced NASH.

ALA undergoes series of complicated desaturation and chain elongation pathway and gets converted into biologically active LC n-3 PUFA. High intake of LA and hence high LA:ALA ratio could inhibit the conversion of ALA. Studies have suggested that substituting LA with ALA would be the most appropriate way of optimizing the conversion of ALA to LC n-3 PUFA^69^. In the present study, substituting LA with ALA (n-6: n-3 ratio of 2) has been shown to increase the incorporation of LC n-3 PUFA at the expense of LC n-6 PUFA in liver phospholipids, suggesting the competitive interaction and inhibition of the desaturation and elongation of LA to LC n-6 PUFA and preferential conversion of ALA to LC n-3 PUFA.

In conclusion, the results of the present study demonstrated that substitution of dietary linoleic acid with α-linolenic acid (n-6:n-3 ratio of 2) or LC n-3 PUFA (n-6:n-3 ratio of 5) protects against the development of Western diet induced NASH as evidenced by improved liver histology, decreased liver and plasma lipids and reduced plasma aminotransferase levels. The protective effect of n-3 PUFA supplementation was attributed to the marked reduction in hepatic oxidative stress and proinflammatory cytokines. The present study also highlights the importance of balancing the n-6 and n-3 PUFA in the diet through suitable blending of vegetable oils for the prevention and management of diet related chronic diseases including NAFLD.

## Electronic supplementary material

Primer sequence used for real time PCR (Supplementary S1)
