# Supplementary material for: Substitution of linoleic acid with α-linolenic acid or long chain n-3 polyunsaturated fatty acid prevents Western diet induced nonalcoholic steatohepatitis
Source: Sci Rep. 2018 Jul 19;8:10953. doi: 10.1038/s41598-018-29222-y (PMC6053361; doi:10.1038/s41598-018-29222-y)
Supplement: Supplementary file 1 — Primer sequence used for real time PCR (Supplementary S1) [file 41598_2018_29222_MOESM1_ESM.pdf]

Substitution of linoleic acid with α-linolenic acid or long chain n-3 polyunsaturated fatty acid prevents Western diet induced non-alcoholic steatohepatitis

Sugeedha Jeyapal[1], Suryam Reddy Kona[1], Surekha Mullapudi Venkata[2], Uday Kumar Putcha[2], Puvaneswari Gurumurthy[1], Ahamed Ibrahim[1]*

Primer sequence used for real time PCR (Supplementary S 1)

Gene Name	Genbank Accession Number	Forward Primer (5'-3')	Reverse Primer (5'-3')
**SCD-1**	**NM_139192.2**	AAGGTGCCCCTCTATCTGGAA	AAATATCCCCCAGAGCAAGGTG
**PPAR α**	**NM_013196.1**	TCCTCTGGTTGTCCCCTTGA	CAGTCTTGGCTCGCCTCTAA
**ACC α**	**NM_022193.1**	ACAACGCAGGCATCAGAAGA	AGCGCTCACATAACCAACCA
**ACOX-2**	**NM_145770.2**	CTTGATCCGGAAGGATGCCA	TGCTTCTCGGTCCCAAATCC
**FAS**	**NM_017332.1**	CCACCGCCTACTACTCCTTA	CTGCTCAAACGATGTGTCTC
**SREBP-1c**	**NM_001276707.1**	CGCTACCGTTCCTCTATCAATGAC	AGTTTCTGGTTGCTGTGCTGTAAG
**CHREBP**	**NM_133552.1**	CGGGGTCGTGTAGACAACAA	CTGACAAGTCCGTGCAGAGT
**PPAR γ**	**NM_013124.3**	AGGCCGAGAAGGAGAAGCTGTTG	TGGCCACCTCTTTGCTCTGCTC
**CPT-1**	**NM_031559.2**	GCAGCTCGCACATTACAAGG	CTCTGCTCTGCCGTTGACTT
**HO-1**	**NM_012580.2**	AGGCTTTAAGCTGGTGATGGC	TGGCTGGTGTGTAAGGGATG
**Collagen α1**	**NM_053304.1**	GATGGACTCAACGGTCTCCC	CGGCCACCATCTTGAGACTT
**Leptin**	**NM_013076.3**	CCGCACGATTCCATGAGCACTAGG	TGGGCCACAGTGAAACACGC
**TNF α**	**NM_012675.3**	ATACACTGGCCCGAGGCAACAC	ACAGACACCGCCTGGAGTTCTG
**IL-1β**	**NM_031512.2**	GCTTCCTTGTGCAAGTGTCTG	GTCGAGATGCTGCTGTGAGA
**IL-6**	**NM_012589.2**	AGGAGTGGCTAAGGACCAAGACC	CTGACCACAGTGAGGAATGTCCAC
**IL-10**	**NM_012854.2**	TGATGCCCCAGGCAGAGAACCA	TCGATGACAGCGTCGCAGCT
